# Diagnosis of parapneumonic pleural effusion with serum and pleural fluid Activin A

**DOI:** 10.1016/j.clinsp.2022.100133

**Published:** 2022-11-11

**Authors:** Guanghui Zhou, Kan Liu, Xiuhai Ji, Yan Fen, Yinjie Gu, Hui Ding

**Affiliations:** a Department of Pulmonary & Critical Care Medicine, The Affiliated Yixing People's Hospital of Jiangsu University, Yixing, China; b Department of Oncology, Affiliated Taicang Hospital of Traditional Chinese Medicine, Taicang, China

**Keywords:** Activin A, Pleural fluid, Serum, Parapneumonia

## Abstract

**Objective:**

The aim is to evaluate the diagnostic value of Activin A levels in serum and pleural fluid on Parapneumonic Pleural Effusion (PPE).

**Methods:**

The authors collected serum and pleural fluid from 86 PPE and 37 Non-PPE (NPPE) patients. Including Activin A, levels of biomarkers such as Lactate Dehydrogenase (LDH), Procalcitonin (PCT), and C-Reactive Protein (CRP) were measured. All factors were calculated for association with days after admission. The diagnostic potential of biomarkers on PPE was considered by Receiver Operating Characteristic (ROC) curve analysis.

**Results:**

Levels of Activin A in serum and pleural fluid of PPE patients were significantly higher than those of the NPPE patients. Moreover, concentrations of Activin A in pleural fluid showed a more obvious relevant days after admission. ROC curve analysis found that Activin A in pleural fluid had AUCs of 0.899 with 93% sensitivity and 84% specificity for PPE diagnosis.

**Conclusion:**

Activin A in pleural fluid correlated with disease severity could act to diagnose PPE.

## Highlights

• Higher concentrations of Activin A in pleural effusion were associated with days of admission in PPE patients.
• Elevated levels of Activin A in pleural effusion exhibited 93% sensitivity and 84% specificity in the diagnosis of PPE.
• Pleural fluid Activin A level had higher AUC and better accuracy in the diagnosis of PPE.

## Introduction

Approximately, pleural fluid concomitantly appeared in 40% of patients with pneumonia.^1^ The timely and accurate diagnosis of Parapneumonic Pleural Effusions (PPE) based on pneumonic etiologies is crucially important. Compared with patients without PPE, pneumonia patients with PPE are suffering from higher mortality and longer hospital stays.^2^ Besides suitable antibiotics, systemic treatment and essential drainage intervention are frequently needed in PPE patients. So, it's critical to earlier investigate the presence and pathogen of PPE in order to take corresponding measures.

Traditionally, it's necessary to distinguish exudate and transudate of pleural fluid via the quantifying ratio of Lactate Dehydrogenase (LDH) and album in the pleural fluid to serum for Light's Criteria Rule.^3^ Moreover, the considered factors of PPE involve neutrophilic exudates with low glucose, elevated LDH, Procalcitonin (PCT), C-Reactive Protein (CRP), and pleural fluid pH. These actors are used clinically with multiple sensitivities and specificities, but none of them are impeccable.^4^

As a foremost member of the Transforming Growth Factor-β (TGF-β) superfamily, activin A was recognized in several physiological functions including inflammation and tissue repair.^5^ Nowadays, activin A was described as proinflammatory and anti-inflammatory actions that participated in the immune response to diseases.^6^ Sideras et al. found overexpression of activin A-induced pathological variation of Acute Lung Injury (ALI)/Acute Respiratory Distress Syndrome (ARDS) in vivo. Furthermore, the therapeutic neutralization of activin-A attenuated the pathogenicity of ALI.^7^ Indeed, activin A was confirmed as the activator of Interleukin 6 (IL-6), Tumor Necrosis Factor-α (TNF)-α in acute inflammation.^8^ Conversely, activin A plays anti-inflammatory action by suppressing IL-6-mediated signaling and secreting Interleukin 1 (IL-1) receptor antagonists.^9^^,^^10^ Functionally, activin A as a remarkable biological actor in asthma, Chronic Obstructive lung Disease (COPD) and lung cancer were supported in recent studies.^11^^,^^12^ In the authors’ previous study, the authors found that levels of serum Activin A were increased in COPD patients with skeletal muscle wasting.^13^ Furthermore, the authors recently identified that higher expression of serum Activin A was associated with poor prognosis of Community-Acquired Pneumonia (CAP) (not yet published). Not surprisingly, the clinical roles of Activin A in pulmonary infectious diseases are needed to be paid attention to. However, the investigation of Activin A in pleural fluid is unclear.

In the present study, the authors aimed to evaluate the clinical value of Activin A level in serum and pleural fluid on the diagnostic potency of PPE.

## Materials and methods

### Study population

This retrospective study was carried out between Jan 2018 and Feb 2021 at Yixing people's hospital affiliated Jiangsu University. This study was approved by the Ethics Committee of Yixing People's hospital (20200022) and complied with the ethical standards of the Declaration of Helsinki. And all participants consent to participate in the study with their written names. 86 patients diagnosed with PPE aged ≥ 18 years were enrolled. And enrolled subjects were excluded from the following criteria, (1) Age < 18 years; (2) Pregnancy; (3) Malignant tumor; (4) Serious renal or heart or liver diseases. 37 patients with Non-Parapneumonic Effusion (NPPE) were also selected for the present study. All participators were categorized as PPE and NPPE groups.

### Data collected

The authors recorded the related clinical characteristics and laboratory parameters from peripheral venous blood and pleural fluid within 24h after admission. Basic parameters including White Blood Cell Counts (WBC), CRP, PCT and LDH were collected.

### Definition of PPE

PPE as a type of pleural lesion was diagnosed according to the criteria.^14^ In this study, enrolled patients without PPE were classified as NPPE, including malignant pleural effusion, transudate pleural effusion and other pathogenesis.

### Assessment of Activin A in blood and pleural fluid

The peripheral venous blood between 6 am and 7 am and pleural fluid within 24h after admission was prepared to check the concentrations of Activin A by using Enzyme-Linked Immunoassays (ELISA) kits (R&D Systems, USA) according to the manufacturer's recommendations.

### Statistical analysis

Categorical variables between groups were compared using by $\chi_{2}$ test via SigmaStat software. Receiver Operating Characteristic (ROC) curves were presented. The Univariate analyses of continuous variables were performed using the nonparametric Mann-Whitney *U* test.

## Results

### Characteristics of enrolled patients

The authors enrolled 86 patients diagnosed with PPE and 37 with NPPE. Among the NPPE group, 16 were confirmed as malignancy pleural fluid with pleural cytology report, 17 were diagnosed with transudate pleural fluid and 4 were linked to other pathogenesis. Table 1 showed the baseline characteristics of the two groups. There were no significant differences in clinical presentation, including age, gender, smoking, blood pressure, respiratory rate, heart rate, and so on.

**Table 1:** Characteristics of enrolled patients with parapneumonic and non-parapneumonic pleural effusion.

	PPE	NPPE	p-value
	n = 86	n = 37	
Gender (male), n (%)	24 (27.9%)	13 (35.1%)	0.34
Age (years old)	62 (52, 76)	63 (51, 74)	0.65
Smoking	37 (43%)	20 (54%)	0.21
Fever episode, n (%)	48 (55.8%)	22 (59.5%)	0.18
Systolic blood pressure (mmHg)	164 (102, 196)	171 (112, 197)	0.23
Diastolic blood pressure (mmHg)	87 (78, 108)	92 (81, 120)	0.34
Pulse rate (times/minute)	92 (72, 121)	94 (63, 127)	0.42
Respiratory rate (times/minute)	17 (15, 22)	19 (17, 23)	0.37
Days of admission (days)	9 (7, 14)	13 (11, 17)	0.11
In hospital mortality, n (%)	4 (4.6%)	2 (5.4%)	0.28

PPE, Parapneumonic Effusion; NPPE, Non-Parapneumonic Effusion.

### Factors in serum and pleural fluid

**Fig. 1:**
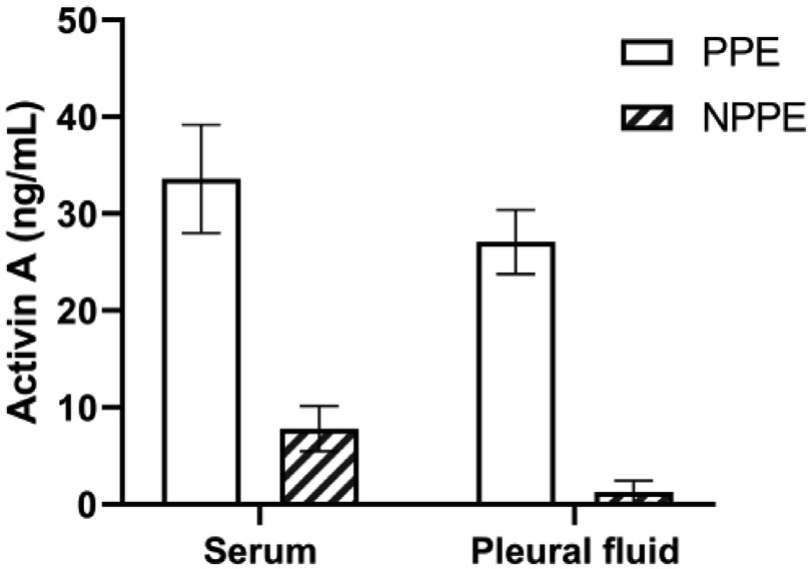
Levels of Activin A in parapneumonia pleural effusion and non-parapneumonia pleural effusion patients. Increased concentrations of Activin A were confirmed in serum and pleural effusion of PPE patients more than those of NPPE patients. PPE, Parapneumonic Effusion; NPPE, Non-Parapneumonic Effusion. *p < 0.05.

The authors investigated the common biomarkers including WBC counts, CRP, PCT, and LDH in a blood sample, and found that there were differences between PPE and NPPE patients. Moreover, inflammatory factors including WBC counts and LDH in the pleural fluid were also significantly different between the two groups (Table 2). Furthermore, Fig. 1 showed that the higher levels of Activin A were present not only in serum but also in PPE, which indicated the closed association between Activin A and PPE. In order or deeply understand the clinical value of Activin A, the authors evaluated the biomarkers in correlation with days after admission in PPE patients. With age and gender adjustments, the authors confirmed that only concentrations of Activin A in the pleural fluid were associated with days of admission in PPE patients (Table 3).

**Table 2:** Levels of biomarkers in patients with parapneumonic and non-parapneumonic pleural effusion.

	PPE	NPPE	p-value
	n = 86	n = 37	
**Serum**			
WBC (× 109/L)	16.1 (10.5, 20.4)	8.6 (4.3, 13.2)	0.005^a^
n (%)	82.5 (72.1, 87.3)	77.6 (62.3, 83.6)	0.18
CRP (mg/L)	173 (137.3, 268.4)	64.2 (13.2, 121.8)	< 0.001^a^
PCT (ng/mL)	0.38 (0.17, 2.97)	0.07 (0.03, 0.23)	< 0.001^a^
LDH (U/L)	103 (78.6, 142.3)	110 (88.1, 178.2)	0.203
Activin A (ng/mL)	33.6 (9.31, 50.7)	7.82 (0.71, 17.11)	< 0.001^a^
**Pleural effusion**			
WBC (× 106/L)	2.35 (1.31, 10.21)	0.42 (0.11, 0.87)	< 0.001^a^
n (%)	81.4 (57.2, 88.5)	11.4 (3.7, 24.8)	< 0.001^a^
CRP (mg/L)	102 (85.6, 174.9)	32.7 (8.41, 82.44)	< 0.001^a^
PCT (ng/mL)	0.33 (0.09, 1.02)	0.05(0.02, 0.13)	< 0.001^a^
LDH (U/L)	326 (147, 628)	76.3 (48, 151.3)	< 0.001^a^
Activin A (ng/mL)	27.1 (8.66, 30.3)	1.29 (0.33, 8.06)	< 0.001^a^

PPE, Parapneumonic Effusion; NPPE, Non-Parapneumonic Effusion; WBC, White Blood Cell; CRP, C-Reactive Protein; PCT, Procalcitonin; LDH, Lactate Dehydrogenase.

a p < 0.05.

**Table 3:** The correlation between days of admission and inflammatory biomarkers in serum and pleural fluid in parapneumonic effusion patients.

	Serum						Pleural effusion					
	WBC	n (%)	CRP	PCT	LDH	Actvin A	WBC	n (%)	CRP	PCT	LDH	Actvin A
***r***	-0.203	-0.34	-0.26	0.504	0.263	0.682	-0.121	-0.252	-0.092	0.466	0.358	0.852
**p-value**	0.392	0.28	0.274	0.013^a^	0.245	0.003^a^	0.613	0.351	0.03^a^	0.026^a^	0.121	< 0.001^a^

*r*, Pearson correlation coefficient; PPE, Parapneumonic Effusion; NPPE, Non-Parapneumonic Effusion; WBC, White Blood Cell; CRP, C-Reactive Protein; PCT, Procalcitonin; LDH, Lactate Dehydrogenase.

a p < 0.05

### Predictive roles of Activin A in serum and pleural on PPE

**Fig. 2:**
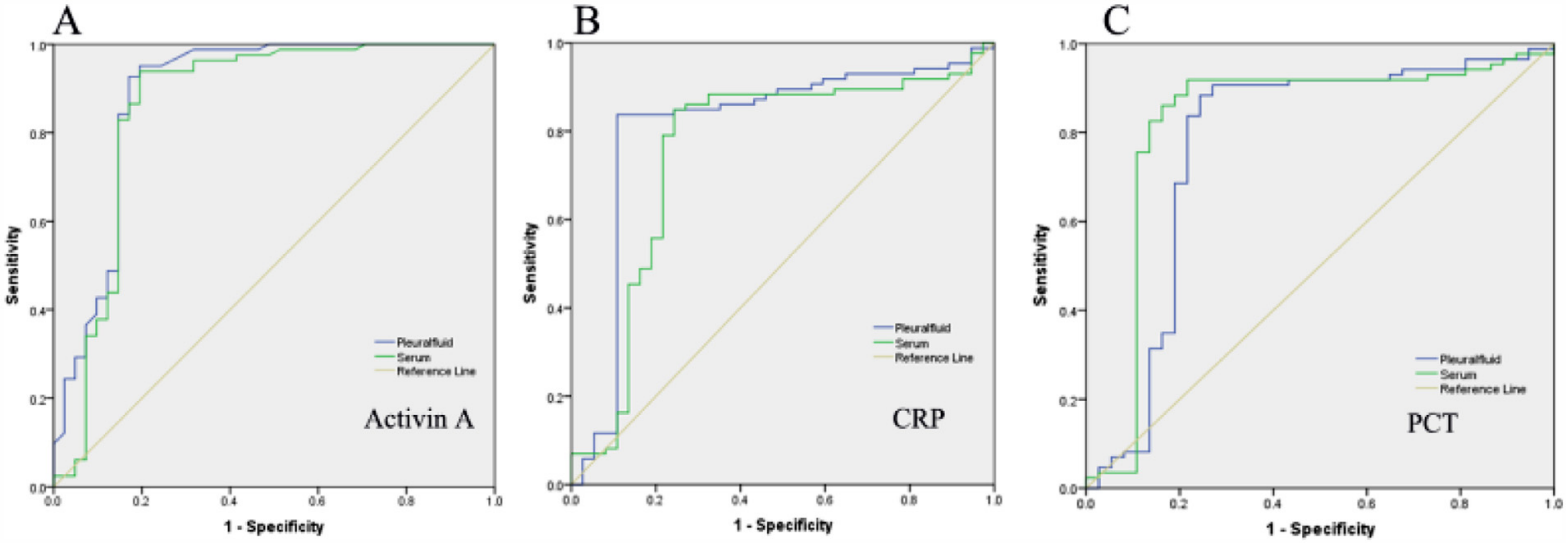
Receiver Operating Characteristic (ROC) curve of biomarkers in the diagnosis of parapneumonic pleural effusion. (A) The diagnostic role of Activin A in serum and pleural effusion on PPE. (B) The ROC of CRP in the diagnosis of PPE. (C) The ROC of PCT in the diagnosis of PPE. CRP, C-Reactive Protein; PCT, Procalcitonin.

It's necessary to earlier definite PPE associated with clinical options and prognosis. The authors evaluated the efficacy of Activin A in predicting PPE by assessing the ROC curve. Fig. 2 showed that Activin A levels in pleural fluid exhibited 93% sensitivity and 84% specificity in the diagnosis of PPE. Table 4 showed that pleural fluid Activin A had an AUC of 0.899 (95% Confidence Interval 0.813‒0.965), and serum Activin A had an AUC of 0.862 (95% Confidence Interval 0.733‒0.95). Pleural fluid CRP and PCT had AUC of 0.813 and 0.782, respectively. And blood CRP and PCT had AUC of 0.756 and 0.821, respectively (Table 4). Together, pleural fluid Activin A level had higher AUC and better accuracy in the diagnosis of PPE.

**Table 4:** The diagnostic value of inflammatory biomarkers in parapneumonic fluid.

	AUC		p-value	95% Confidence Interval		Sensitivity	Specificity
				Lower	Upper		
Serum							
CRP	0.756		0.006^a^	0.649	0.860	0.73	0.67
PCT	0.821		0.004^a^	0.722	0.923	0.75	0.74
Activin A	0.862		0.002^a^	0.733	0.950	0.89	0.81
Pleural effusion							
CRP	0.813		0.003^a^	0.711	0.906	0.84	0.74
PCT	0.782		0.001^a^	0.662	0.883	0.86	0.82
Activin A	0.899		< 0.001^a^	0.813	0.965	0.93	0.84

AUC, Area Under Curve; PPE, Parapneumonic Effusion; NPPE, Non-Parapneumonic Effusion; CRP, C-Reactive Protein; PCT, Procalcitonin.

a p < 0.05.

## Discussion

To the best of our knowledge, it's firstly to assess concentrations of Activin A both in serum and in pleural fluid for diagnosis of PPE. In the present study, elevated levels of serum and pleural fluid Activin A appeared in PPE compared with NPPE patients. Furthermore, increased levels of Activin A in the pleural fluid were determined to be associated with days after admission. Pleural fluid Activin A had acceptable sensitivity and specificity to diagnose PPE.

As a consequence of pressure, transudative fluid outcome in the pleural space. Coupled with increased inflammatory cells in the pleura, pleural fluid as an exudative fluid developed with complex etiological factors.^15^ In addition to malignancy, tuberculous-induced pleural fluid was rich in lymphocytes.^16^ However, PPE from bacteria is an abundant supply of neutrophils. Unluckily, morbidity and mortality are higher in patients with pneumonia and pleural fluid than in individuals without pleural fluid.^17^ The radiological and ultrasonic test is commonly accepted to determine pleural fluid. Popular biomarkers of inflammation as CRP and PCT in serum and pleural fluid were used to predict PPE.^18^ Falguera et al. found pleural fluid CRP contributed to the diagnosis and assessment of the severity of PPE.^19^ Although CRP has value in predicting inflammation severity, the non-specific characteristic is regrettable.^20^ The present study also found the clinical effects of CRP on PPE diagnosis with moderate accuracy. In the previous study, PCT concentrations in serum and pleural fluid showed predictive roles in PPE patients.^21^ However, unexpected sensitivity and specificity of PCT in the diagnosis of PPE were concluded in a meta-analysis study.^22^ The present data found levels of PCT in serum levels in serum and in pleural fluid predicted PPE with AUC values (0.821 and 0782). In the present study, the authors firstly confirmed Activin A in serum and pleural fluid had a predictive ability of PPE, with higher AUC values (0.862 and 0.899). Previous studies confirmed the abnormal expression of Activin A in serum was found in various pulmonary diseases.^23^^,^^24^ Consisted of lower cut-off values of CRP and PCT, concentrations of Activin A in pleural fluid not in serum could be more suitable for diagnosing PPE with better sensitivity and specificity.

Activin A was proven to participate in the development of pulmonary inflammation.^25^ The biological functions of Activin A as a key regulator of other inflammatory cytokines including TNF-α, Interleukin-1β (IL-1β), and Interleukin-6 (IL-6) were confirmed in inflammatory response.^26^^,^^27^ The clinical investigation found that a higher concentration of Activin A in Bronchoalveolar Fluid (BALF) could predict poorer mortality in ARDS patients.^28^ The authors’ previous study found that compared with healthy individuals, patients with the chronic obstructive pulmonary disease have elevated levels of serum Activin A correlated with TNF-α expression.^13^ Furthermore, the authors recently revealed that increased levels of Activin A in serum not influenced by etiology play a more effective predictor of hospital mortality in CAP patients (not yet published). However, to our knowledge, there's no associated report about Activin A in pleural fluid with pulmonary inflammation, especially with PPE. The forecast role of Activin A in pleural fluid is unclear. It's necessary to observe the clinical value of Activin A in PPE.

Once diagnosed, a therapeutic strategy including enough antibiotics and proper drainage is necessary. Patients with severe cases of PPE always suffered from longer days after admission with standard treatment regimens. Therefore, it's essential to proactively determine the severity of their condition, in order to identify patients who would need aggressive interventions earlier. In this study, all PPE patients were made in drainage as standard therapy. In order to evaluate the association between Activin A and disease severity, the authors used the number of days after admission to represent the severity of PPE. The data of the present study confirmed that Activin A in pleural fluid correlating with days of admission is just an appropriate indicator of severity in PPE.

As a retrospective study, the present study faced some limitations. First, the numbers of NPPE patients are fewer, and it's difficult to classify subgroup analyses with the different pathogen of pleural fluid. Second, meanwhile, the authors have not compared the levels of Activin A in BALF, serum, and pleural fluid. Third, levels of Activin A in pleural fluid at different time points after admission were unknown.

The present study revealed that Activin A concentration in pleural fluid exhibited a capacity for diagnosis of PPE and for gauging the severity of the disease. The present data supported a close relationship between levels of Activin A and local inflammation in the pleura. Whether it is suitable to distinguish PPE from multiple etiologies of pleural fluid needs further investigation.

## Authors' contributions

Liu K, Zhou G and Ji X prepared the samples and carried out the data analysis. Fen Y collected samples. Gu Y and Ding H designed the project. Ding H wrote the manuscript.

## Declaration of Competing Interest

The authors declare no conflicts of interest.
